# The Real-World Effectiveness of Human Immunodeficiency Virus Pre-Exposure Prophylaxis in Adults in Alberta, Canada: A Retrospective Population-Based Cohort Study

**DOI:** 10.1155/cjid/9340622

**Published:** 2025-08-10

**Authors:** Elissa Rennert May, Mu Lin, Shannon L. Turvey, Derek S. Chew, Marcello Tonelli, Scott Klarenbach, Neesh Pannu, Emily Christie, Stephanie Thompson, Aminu Bello, Darren Lau, Caley B. Shukalek, Raynell Lang, David Collister

**Affiliations:** ^1^Division of Infectious Diseases, Department of Medicine, University of Calgary, Calgary, Alberta, Canada; ^2^Alberta Strategy for Patient Oriented Research Unit, Provincial Research Data Services, Alberta Health Services, Edmonton, Alberta, Canada; ^3^Division of Infectious Diseases, Department of Medicine, University of British Columbia, Vancouver, British Columbia, Canada; ^4^Department of Cardiac Sciences, University of Calgary, Calgary, Alberta, Canada; ^5^Division of Nephrology, Department of Medicine, University of Calgary, Calgary, Alberta, Canada; ^6^Division of Nephrology, Department of Medicine, University of Alberta, Edmonton, Alberta, Canada; ^7^Division of General Internal Medicine, Department of Medicine, University of Alberta, Edmonton, Alberta, Canada; ^8^Division of General Internal Medicine, Department of Medicine, University of Calgary, Calgary, Alberta, Canada

## Abstract

**Background:** In clinical trials, pre-exposure prophylaxis (PrEP) with tenofovir disoproxil fumarate-emtricitabine (TDF/FTC) or tenofovir alafenamide-emtricitabine (TAF/FTC) is up to 99% efficacious in preventing human immunodeficiency virus (HIV) infection. The real-world effectiveness of PrEP has not been extensively evaluated in Canada.

**Methods:** This population-based cohort included adults without HIV as determined by viral serology and ICD-9/ICD-10 codes from Alberta with ≥ 3 months of PrEP prescriptions. It used provincial administrative data. Patients were followed from their first PrEP prescription until diagnosed HIV infection or censoring. Cox proportional hazard models were used to identify independent predictors of HIV infection.

**Results:** A total of 4750 adults with a mean (SD) age of 35.9 (11) years of which 8% were female were prescribed PrEP including TDF/FTC (97.5%) or TAF/FTC (2.5%). There were 335 HIV infections (92.9% effectiveness) over median cohort follow-up of 1.0 years (IQR 1.9) with 4.89 (95% CI 4.38, 5.44) HIV infections per 100 patient years. Age (HR 1.04, 95% CI 1.03–1.05 per 1 year increase), male sex (HR 0.34, 95% CI 0.27–0.44), CKD Stage G3 (HR 2.39, 95% CI 1.82, 3.14), SES (4th and 5th quintiles versus 1st quintile), drug use (HR 2.11, 95% 1.45, 3.08), and history of STI (HR 0.45, 95% CI 0.29, 0.72) were independent predictors of HIV infection. The HIV incidence decreased to 1.5 (95% CI 1.2, 1.8) and 0.6 (95% CI 0.4, 0.9) per 100 patient years in cohorts with negative baseline HIV serology with 180 and 30 days prior to index PrEP prescription.

**Conclusion:** HIV PrEP appears to be effective for preventing HIV infection in this real-world population-based study in Alberta, Canada. Strategies to mitigate residual HIV risk in PrEP users are needed.

## 1. Introduction

Pre-exposure prophylaxis (PrEP) with tenofovir disoproxil fumarate-emtricitabine (TDF/FTC) or tenofovir alafenamide-emtricitabine (TAF/FTC) is safe and efficacious for preventing human immunodeficiency virus (HIV) infection [1–6]. PrEP is used in individuals who are HIV-negative but at a high risk of acquiring the virus. Eligible patients may include men who have sex with men (MSM), transgender women, or gender diverse people with risk factors with exposure-prone sexual activities, heterosexual persons with HIV-positive sexual partners that are not virally suppressed or with exposure-prone sexual activities, and people who inject drugs (PWID) [7–9].

In Canada, PrEP consists of TDF/FTC (300 mg/200 mg) [10] or TAF/FTC (25 mg/200 mg) [11] in individuals with estimated glomerular filtration rate (eGFR) 30–60 mL/min/1.73 m^2^, but TAF/FTC is not recommended for HIV prevention in the setting of receptive vaginal sex or event-driven/on-demand/pill-in-pocket strategies for adults who are male assigned sex at birth. In 2021, PrEP prevalence ranged from 16 to 108 per 100,000 persons across Canada with a mean prevalence of 70 per 100,000 of which 98% of users were male and 70% were prescribed by primary care physicians [12].

When taken as prescribed in adherent populations in randomized controlled trials (RCTs), PrEP is up to 99% efficacious in preventing HIV, has no major safety concerns, and has a low incidence rate of drug-resistant HIV [13]. However, real-world evidence for the effectiveness of PrEP is limited in Canada. Assessment of real-world PrEP effectiveness is important as real-world populations of persons who might benefit from PrEP are typically more diverse than those represented in clinical trials [14, 15]. In addition, PrEP is associated with an increased risk of adverse kidney events (typically mild and reversible kidney injury) but less than 1% of participants in pivotal clinical trials had an estimated creatinine clearance (eCrCl) < 60 mL/minute [16], so its efficacy and safety in the chronic kidney disease (CKD) population is uncertain. The objective of this study was to determine the real-world effectiveness of PrEP in a Canadian provincial public health system using administrative data and to determine independent predictors for HIV infection in incident PrEP users. We were specifically interested in how CKD [17] may impact the success of PrEP at preventing HIV infections in adults given its kidney clearance which may modify its pharmacokinetics and toxicity.

## 2. Methods

### 2.1. Study Design

We conducted a population-based retrospective cohort study in Alberta, Canada, using linked administrative data. We included all adults (age ≥ 18 years) who were prescribed PrEP with TDF/FTC or TAF/FTC for ≥ 30 days from April 1, 2013, to March 31, 2022. Follow-up was from the date of dispensed PrEP prescription until loss to follow-up from the databases, migration from Alberta, death, or the end of the study period. Individuals who discontinued PrEP without further continuous prescriptions were censored at their last prescription plus the number of days of supply. We did not include individuals who stopped and restarted PrEP and only focused on the index PrEP usage period in the study. Ethics approval was obtained from the Health Research Ethics Board at the University of Alberta (Pro00121194). We followed the STROBE reporting guidelines.

### 2.2. PrEP Prescribing and Monitoring

In Alberta, PrEP with FTC/TDF has been available as part of the Alberta Health Care Insurance Plan since October 1, 2018, when prescribed by a PrEP provider with appropriate training. Previously, individuals had to pay out-of-pocket or obtain reimbursement through drug insurance plans [18] while FTC-TAF is only covered by private insurance or compassionate supply. Alberta PrEP eligibility criteria has been consistent with Canadian guidelines [7] and includes MSM, transgender women, and gender diverse people who have had condomless anal sex within the last six months with the following risk factors: (1) a bacterial sexually transmitted infection (STI) in the last 12 months; (2) nonoccupational post-exposure prophylaxis more than once; (3) ongoing sexual relationship with an HIV-positive partner with a viral load > 40 copies/mL or whose HIV status was unknown and was an MSM or PWID; or (4) had an HIV Incidence Risk Index for Men who have Sex with Men (HIRI-MSM) score ≥ 11 [19]. Eligibility also includes heterosexual people and PWID with a sexual or drug-sharing HIV-positive partner with a viral load > 40 copies/mL or whose HIV status was unknown and was a MSM, PWID, or from a country with high HIV prevalence [20].

The Alberta PrEP guidelines recommend baseline testing as follows: serology for hepatitis A, B, and C, HIV (to rule out pre-existing HIV prior to PrEP due to the risk of resistance and treatment failure), and syphilis; serum creatinine; and nucleic acid amplification tests (NAAT) testing from urogenital and/or extragenital sites for chlamydia and gonorrhea. After initiation of PrEP, 1-month follow-up recommendations include repeat serology for HIV and serum creatine with repeat screening of HIV, syphilis, chlamydia, gonococcal, and serum creatinine recommended every 3 months. Serum creatinine testing varies across the province but include enzymatic or nonenzymatic assays referenced to IDMS standards with eGFR calculated using the 2012 CKD-EPI equation [21].

### 2.3. Eligibility Criteria

Inclusion criteria were the following:

1. Adult males and females 18 years of age or older
2. Alberta Health Care Insurance Plan coverage
3. HIV-negative determined by the absence of ICD-9/ICD-10 codes [22] and negative viral serology if performed within −365 days to +30 days of the index date
4. TDF/FTC or TAF/FTC prescription for ≥ 1 month

Exclusion criteria were the following:

1. Missing date of birth or sex
2. Less than 365 days of observation time prior to the index date
3. No baseline creatinine and eGFR within 180 days of the index date
4. Viral hepatitis B including administrative data with ICD-9/ICD-10 codes or previous positive viral hepatitis B serology (HBV surface antigen, core antibody, E antigen, E antibody, viral DNA) if performed within 365 days of index date
5. Kidney failure receiving maintenance dialysis or kidney transplant within 365 days of the index date
6. Missing material deprivation index (a marker of SES)

A list of the applicable administrative codes used to define the eligibility criteria is provided in Appendix Table 1. Participants were not required to specifically have negative HIV serology (HIV antibody or antigen or HIV RNA) prior to the index date to be included in the cohort, but this was explored in sensitivity analyses.

### 2.4. Data Sources

Administrative data was obtained from the Alberta Strategy for Patient Oriented Research Unit (AbSPORU database) which is housed at Alberta Health Services [23]. Patient records in the repository are deidentified using an encrypted personal health information number (ULI) as a quasi-identifier to protect privacy. A summary of included databases is provided in Appendix Table 2.

### 2.5. Exposures, Covariates, and Outcome

The study exposure was a prescription for PrEP with either TDF/FTC or TAF/FTC ≥ 30 days at any time during the study period. Gaps between PrEP prescriptions were not permitted and resulted in censoring. Prescriptions were ascertained via anatomic therapeutic classification (ATC) codes as defined in Appendix Table 3. Covariates measured to describe baseline characteristics of the study population include demographics (age, sex assigned at birth), socioeconomic status (material deprivation index), geography (urban vs. rural), comorbidities within the last 2 years prior to the index date [hypertension, diabetes, heart failure, stroke, myocardial infarction (MI), peripheral vascular disease, history of drug use, gonorrhea, chlamydia, syphilis, and STI with relevant ICD-9 and ICD-10 codes [24] are provided for each comorbidity in Appendix Table 4], laboratory testing (serum creatinine, eGFR, HIV), serology (laboratory-tested antigen, antibody, or RNA), HBV serology (HBsAg, HBcAb, HbeAg, HBV DNA), STI testing including gonorrhea and chlamydia NAAT as well as syphilis enzyme immunoassay (but not rapid plasma reagin or venereal disease research laboratory test) within the last 365 days prior to the index date (positive/reactive vs. negative/nonreactive/indeterminate), and medications (ACE inhibitors, ARBs, SGLT2 inhibitors, NSAIDs including COX-1 and COX-2 inhibitors). CKD was defined as two eGFR values < 60 mL/min/1.73 m^2^ and at least 90 days apart. CKD was classified by KDIGO staging based on eGFR but no albuminuria [17]. The primary effectiveness outcome was HIV infection defined by positive HIV serology or HIV RNA. HIV testing in Alberta is done with a fourth-generation HIV Ag/Ab Combo EIA (Abbott ARCHITECT, Abbott, Chicago, Illinois, USA); if positive, it is repeated, and if positive again, it is followed by a confirmatory test (Geenius HIV 1/2 Antibody Differentiation Assay, Bio-Rad, Montreal, QC, Canada).

### 2.6. Statistical Analysis

Baseline characteristics for the entire cohort, TDF/FTC users, TAF/FTC users, and by CKD stages are summarized using descriptive statistics. Continuous variables are expressed as mean and standard deviation or median and interquartile range (IQR) depending on the normal versus non-normal distribution. Categorical variables are expressed as frequencies and percentages. TDF/FTC and TAF/FTC users are compared using the *t*-test/Mann–Whitney *U* test or the Chi-squared test when appropriate.

To compare the association of PrEP with the incidence of HIV infection, the number of events and follow-up times for all patients were collected to calculate incidence rates reported per 100 person years of exposure. Incidence rates were reported by the presence or absence of PrEP medication, and by each PrEP medication individually.

Multivariable Cox proportional hazard models were adjusted for demographics, comorbidities, medications, and laboratory values [25]. We did not adjust for adherence and assumed that continuous PrEP dispensation reflected an adherent population. For all models, covariates associated with outcomes were included without stepwise selection or interactions. The proportional hazards assumption was tested using the Supremum test. Outliers and collinearity were assessed using likelihood displacement scores > 0.25 and correlation coefficients > 0.05, respectively. The primary analysis limited the cohort to individuals with negative HIV serology testing within the 30 days prior to inclusion, and sensitivity analyses were performed limiting the cohort to individuals with negative HIV serology testing 180 days and 365 days without any previous HIV ICD-9/ICD-10 codes or positive HIV serology testing (but not necessarily a previous negative HIV test) and to vary the possibility of including patients with undiagnosed HIV prior to receiving PrEP and censoring at the last HIV test instead of PrEP discontinuation. A logistic regression model adjusted for the same covariates over the total duration of follow-up for each participant was completed to limit the effect of informative censoring. Given the construction of this population-based cohort, there was no missing data for any covariate. *p* values < 0.05 (two-tailed) were considered statistically significant. All analyses were done with SAS software, version 9.4 (SAS Institute, Cary, NC).

## 3. Results

**Figure 1:**
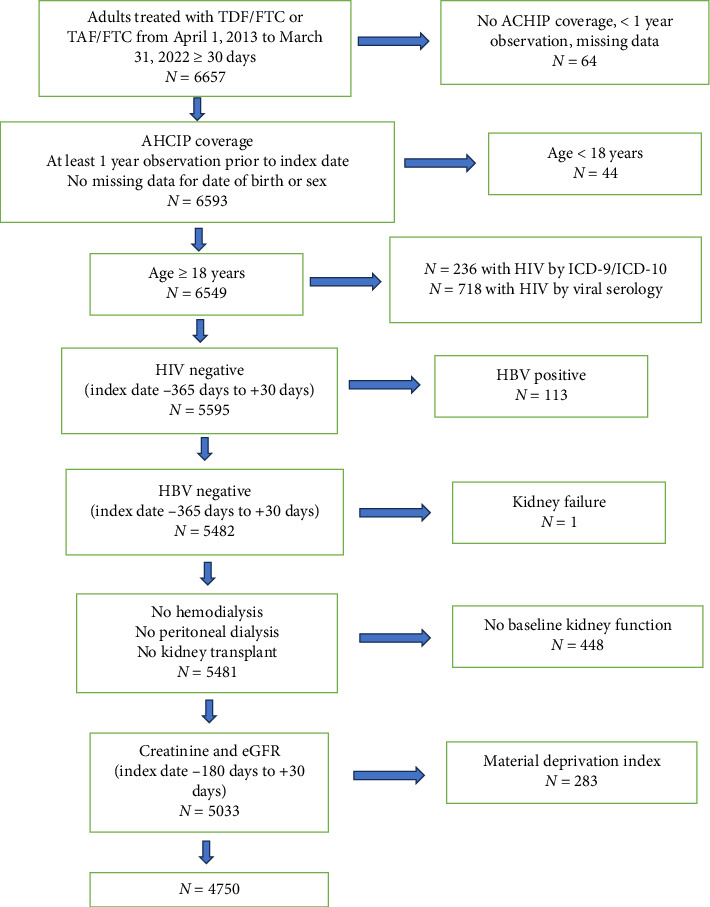
Participant flow diagram. *Note:* TDF/FTC = tenofovir disoproxil fumarate, TAF/FTC = tenofovir alafenamide, ACHIP = Alberta Health Care Insurance Plan, HIV = human immunodeficiency virus, ICD = international classification of diseases, HBV = viral hepatitis B, and eGFR = estimated glomerular filtration rate.

The cohort included 4750 adults prescribed PrEP (97.5% TDF/FTC and 2.5% TAF/FTC) with a mean (SD) age of 35.9 (11) years, and 8.2% were female sex (Figure 1). The median duration of PrEP coverage was 1.0 years (IQR 1.9) [1.0 years (IQR 1.9) and 0.3 years (IQR 1.3) respectively for TDF/FTC and TAF/FTC]. In the 4750 PreP users, PrEP was discontinued in the setting of 335 HIV infections (7.1%), 13 deaths (0.3%), 19 migrations (0.4%), 2490 with no further continuous prescriptions (52.4%), and 1893 (39.8%) will still using PrEP at the end of the study period.

PrEP was initially prescribed for 30 days in 3125 persons (65.7%), 60 days in 157 persons (3.3%), 90 days in 824 persons (17.3%), and other durations in the remaining 650 persons (13.7%). In follow-up beyond the index prescription, PrEP was most prescribed for 30 days (39.4%), 60 days (4.9%), or 90 days (28.2%). The mean (SD) interval between HIV serology testing over follow-up was 98 (81) days.  Table 1 shows baseline comorbidities of all PrEP users; the most common comorbidities were hypertension (7.9%), diabetes (4.1%), heart failure (2.8%), and depression (11.9%); 9.3% had eGFR > 120 mL/min/1.73 m^2^ (hyperfiltration), 57.4% had eGFR 90–120 mL/min/1.73 m^2^, 26.0% had eGFR of 60–89 mL/min/1.73 m^2^, 7.3% had eGFR 30–59 mL/min/1.73 m^2^, and no patients had eGFR < 30 mL/min/1.73 m^2^.

Adults treated with TAF/FTC, as compared to TDF/FTC, were older, and more likely to have a history of hypertension, diabetes, heart failure, MI, CKD, and be treated with RAAS inhibitors (Table 1). As compared to those without CKD, adults with CKD were older, more likely to be female sex, have a history of hypertension, diabetes, lower SES and receive PrEP with TAF instead of TDF (Table 2). Prior to PrEP prescription, HIV serology was obtained in the previous 30, 60, 90, 180, and 365 days in 3579 (75.3%), 3816 (80.3%), 3928 (81.9%), 4071 (82.7%), and 4141 (87.1%), respectively. HIV serology was obtained in the first 7 days in 285 (6.0%) and in the first 30 days in 1859 (39.1%); 140 (2.9%) had HIV serology done in the first 30 days but not within the last year prior to PrEP prescription.

In the primary analysis limiting the cohort to those with negative HIV serology within 30 days of the index date as recommended by current guidelines with a median follow-up of 0.6 years (IQR 1.1) (*n* = 3579, 30 HIV events, 99.2% effectiveness, incidence of HIV 0.6 (95% CI 0.4, 0.9) per 100 patients years), age (HR 1.05, 95% CI 1.01–1.09 per 1 year increase), male sex (HR 0.26, 95% 0.09–0.76), TDF versus TAF (HR 0.26, 95% CI 0.08–0.88), and geography (urban versus rural HR 0.21, 95% CI 0.08–0.57) were significant predictors of HIV infection (Table 3).

In the sensitivity analysis limiting the cohort to those with negative HIV serology during the 180 days preceding the index date with a median follow-up of 0.8 years (IQR 1.2) (*n* = 4071, 84 HIV events, 97.9% effectiveness, incidence of HIV 1.5 (95% CI 1.2, 1.8) per 100 patient years), PrEP type and geography were no longer statistically significant but CKD status (Stage 3G CKD HR 3.27, 95% CI 1.86–5.73) and drug use (HR 3.41, 95% 1.53–7.64) became significant (Table 4).

In the sensitivity analysis limiting the cohort to those without an HIV ICD-9/10 code or positive HIV serologic test in the last 365 days, there were 335 HIV infections in 4750 individuals (92.9% effectiveness) and the median (IQR) time from PrEP initiation to the diagnosis of HIV infection was 1.1 years (1.0). The Kaplan–Meier survival curve is shown in Appendix Figure 1. Overall, the incidence of HIV was 4.89 (95% CI 4.38, 5.44) per 100 patient years. The incidence of HIV in TDF/FTC users was 4.81 (95% CI 4.30, 5.36) per 100 patient years, and the incidence of HIV in TAF/FTC users was 9.61 (95% CI 4.61, 17.68) per 100 patient years (95% CI 9.61, 17.68). Independent predictors of HIV infection were age (HR 1.04 per year increase, 95% CI 1.03–1.05), male sex (HR 0.34, 95% CI 0.27–0.44), CKD (HR 2.39, 95% CI 1.82–3.14 for eGFR < 60 mL/min/1.73 m^2^, and HR 0.58, 95% CI 0.41–0.81 for eGFR 60–89 mL/min/1.73 m^2^), SES, drug use (HR 2.11, 95% CI 1.45–3.08), and no history of STI (HR 0.45, 95% CI 0.29–0.72) (Table 5). The only predictors that violated the proportional hazards assumption assessed by the Supremum test were CKD stage and drug abuse. Stratified analyses for these covariates did not meaningfully change our results (not shown). Only 3/4750 (0.06%) individuals were outliers with likelihood displacement scores > 0.25 so they were not removed from the analysis. Only sex and history of drug abuse had a correlation > 0.05 at 0.15 suggesting collinearity; removing a history of drug abuse as a covariate from all models did not meaningfully change our results (not shown). The sensitivity analysis which censored at the last HIV test instead of PrEP discontinuation did not meaningfully change our results (Appendix Table 5). The results of the primary analysis did not change when a logistic regression model was performed except for TDF/FTC (OR 2.25, 95% 1.08–5.18) and depression (OR 0.61, 95% CI 0.38–0.94) which become independent predictors of HIV infection (Table 6). The HIV incidence for each subgroup included in each regression model is located in Appendix Table 6.

## 4. Conclusion

In this population-based retrospective observational study of 4750 adults prescribed PrEP in Alberta from April 1, 2013, to March 31, 2022, we found that PrEP was highly effective in preventing HIV infection with effectiveness ranging from 92.9% to 99.2% depending on the population defined the best practice of ensuring baseline HIV serology is completed within 30 days of index PrEP which eliminates pre-existing HIV infection. Surprisingly, PrEP was prescribed without baseline HIV serology within 30 days in more than 1/4 of PrEP users in this cohort, but these patients could have undergone HIV point of care or rapid testing whose results are not available in provincial health databases for both baseline and follow-up testing.

Overall, the cohort was relatively young with a mean age of approximately 35 years and less than 10% had any documented history of hypertension, diabetes, vascular disease, heart failure, or CKD. TAF/FTC users, as compared to TDF/FTC users, were older and more likely to have a history of hypertension, diabetes, heart failure, MI, CKD, and be treated with RAAS inhibitors. Independent predictors of HIV infection were older age, female sex, CKD, lower SES, drug use, and no history of STI. This study expands on a previous retrospective study of 511 adults prescribed PrEP in Alberta from March 2016 to June 2019 from two primary care clinics, four sexual reproductive health clinics, and one STI clinic in urban/semiurban settings that included 98.4% cisgender males with 100% baseline HIV testing with only 1 HIV seroconversion over follow-up [26].

Our finding that PrEP is highly effective in preventing HIV infection is consistent with previous clinical trials and observational studies globally. In a systematic review of randomized trials and observational studies, PrEP was associated with reduced risk of HIV infection compared with placebo or no PrEP (2.37% vs. 4.18%, risk ratio 0.46, 95% CI 0.33–0.66]) [27]. The effectiveness of PrEP varies across real-world settings as shown in Appendix Table 7 which summarizes recent real-world effectiveness studies including noncontrolled trials and prospective/retrospective observational studies. HIV incidence per 100 patient years and effectiveness compared to non-PrEP users varies across studies with heterogeneity due to different populations, indications for PrEP, baseline risk of HIV infection, baseline HIV serology windows pre/post PrEP initiation to exclude patients with pre-existing HIV, adjustment for HIV risk factors, and adherence. The high incidence rate of HIV infection in this cohort, even when limiting to those with negative HIV serology within 30 or 180 days prior to PrEP prescription may be due to many different factors including baseline and longitudinal HIV risk, PrEP adherence, and even local HIV resistance to PrEP. The difference in TDF versus TAF that was only significant in the primary analysis must be interpreted with caution given the limited number of events and potential for residual confounding.

Our findings that independent predictors of HIV infection in PrEP users were older age, female sex, CKD stage G3, lower SES, drug use, and no history of STI are novel and need to be confirmed in further studies. In the Expanded PrEP Implementation in Communities-New South Wales (EPIC-NSW) study which was a pragmatic, prospective, single-arm, implementation study of daily, oral PrEP in 31 sites (sexual health clinics, general practices, and a hospital) from March 2016 to April 2018 and included 9596 HIV-negative adults (99.2% with at least one follow-up HIV test) prescribed PrEP, there were 30 HIV seroconversions over 18,628 person-years with an incidence of 1.61 per 1000 person-years (95% CI 1.13–2.30) [28]. Due to the low number of events, only univariate Poisson regression was performed, which identified younger age, MSM population, history of STI, use of crystal methamphetamines, number of HIV risk factors, and lower mean medication possession ratio as independent risk factors for HIV infection. It is unclear why older age, CKD, and no history of STI were risk factors in our study. Older age may be related to PrEP adherence which we were unable to measure due to our study's focus on PrEP dispensation. The finding that females taking PrEP are at increased risk of HIV infection may be due to lower adherence as a result of behavioral factors or more likely inadequate drug concentrations in the vaginal mucosa compared to the rectum with higher necessary adherence thresholds to achieve target protective concentration (and thus why on-demand PrEP is not recommended), although these thresholds have not been established [29–31]. Prior STI being linked with lower risk of incident HIV infection is surprising but may be due to changes in sexual practices and their associated risks such as specific types of sex and concurrent condom use. CKD being a risk factor is also surprising as decreased kidney clearance would presumably result in elevated PrEP tissue levels and protection but as above, CKD may impact PrEP adherence due to concerns about toxicity or HIV susceptibility due to immunodeficiency [32, 33]. Our finding that TDF/FTC and TAF/FTC are equivalent for HIV PrEP is consistent with previous literature as TAF/FTC has been shown to be noninferior to TDF/FTC with better kidney and bone biomarker safety [5] but increased weight gain and dyslipidemia [34], and, more recently, incident hypertension and statin use [35]. However, the kidney, bone, and cardiovascular safety of TDF/FTC and TAF/FTC in CKD and those with CKD risk factors needs to be evaluated in population-based studies.

Our study has several strengths. The study's uniquely population-based cohort includes a large sample of diverse adult PrEP users with complete data on demographics, SES, comorbidities, medications, and kidney function with minimal attrition and complete follow-up. It is novel because of its exploration of CKD stage G3 as a risk factor for HIV infection in the setting of PrEP. However, it has some limitations. First, we were unable to collect and adjust for ethnicity/race which are independently associated with HIV infection [36, 37] due to restrictions with regard to data privacy and confidentiality. Secondly, we were unable to fully characterize and adjust for participant HIV risk factors and the indication for PrEP prescription (e.g., sexual behaviors, drug use) as this information was not captured in administrative databases. Specifically, we may have inadvertently included individuals with PEP given the duration of PrEP prescription was 30 days, but PEP is typically given for up to 28 days [7] only and therefore the risk of this was fairly minimal. Third, the prescription and dispensation of TDF/FTC and TAF/FTC does not indicate a patient is necessarily adherent and we were unable to differentiate between daily PrEP use and nondaily PrEP use and censored the last prescription duration without consideration of re-initiation. Fourth, the number of events in our sensitivity analyses were quite low with a loss of precision so we were unable to include additional covariates in Cox models due to the risk of nonconvergence or overfitting. We were also unable to include matched control patients (i.e., adults at risk of HIV infection but not receiving PrEP) to compare real-world effectiveness in PrEP users versus PrEP nonusers. We also did not examine the use of long-acting injectable PrEP which was not yet available in Canada [38] during the study period but has recently been approved. We could not examine PrEP prescribing or outcomes by primary care provider, infectious disease specialist, or other providers [39] or clinical setting or delivery models such as telemedicine [40] as these data were not readily available, but the number of approved PrEP providers in Alberta is currently more than 100 prescribers [41]. Fifth did not account for COVID-19-related changes in sexual behavior, access to care, adherence, or HIV testing [42] or other temporal trends related to PrEP coverage/reimbursement in Alberta due to the limited number of events. Censoring occurred at the number of days the last prescription was provided although it is acknowledged that individuals may be protected for several days following PrEP discontinuation [43]. We do not think censoring due to competing risks was an issue given the low death event rate although informative censoring due to lack of PrEP prescription renewal may reflect PrEP intolerance, nonadherence, and/or a change in HIV infection risk that may modify PrEP effectiveness after the last prescription in PrEP discontinuers. Lastly, we do not know how point-of-care testing may have influenced our results which may have potentially led to earlier or later (deferred) laboratory HIV serology testing depending on the presence of true/false positives/negatives.

In conclusion, this population-based retrospective cohort study from a single Canadian province showed that PrEP is highly effective in a real-world setting and nearly approaches the 99% effectiveness seen in RCTs when restricted to those with recent negative baseline HIV serology relative to the index PrEP prescription. We identified potential novel independent predictors for HIV infection in the setting of PrEP including older age, female sex, and CKD stage G3 possibly related to adherence, pharmacology, and immunologic susceptibility which require validation in other cohorts and further mechanistic exploration.

## Figures and Tables

**Table 1:** Cohort baseline demographics by pre-exposure prophylaxis type.

		**TDF/FTC + TAF/FTC (*n* = 4750)**	**TDF/FTC (*n* = 4633)**	**TAF/FTC (*n* = 117)**	**p** **value**

Demographics	Age (mean, SD)	**35.9 (11)**	**35.7 (11)**	**46.2 (14)**	**< 0.01**
	Female sex (*n*, %)	388 (8%)	374 (8%)	14 (12%)	0.20

Geography	Urban	4467 (94%)	4360 (94%)	107 (91%)	0.23

Comorbidities	Hypertension (*n*, %)	**377 (8%)**	**355 (8%)**	**22 (19%)**	**< 0.01**
	Diabetes (*n*, %)	**194 (4%)**	**181 (4%)**	**13 (11%)**	**< 0.01**
	Heart failure (*n*, %)	**12 (0.3%)**	**10 (0.2%)**	**2 (2%)**	**0.03**
	Stroke (*n*, %)	15 (0.3%)	14 (0.3%)	1 (1%)	0.31
	Myocardial infarction (*n*, %)	**8 (0.2%)**	**5 (0%)**	**3 (2%)**	**< 0.01**
	Alcohol misuse (*n*, %)	68 (1%)	66 (1%)	2 (2%)	0.68
	Depression (*n*, %)	559 (12%)	548 (12%)	11 (9%)	0.56
	Drug use (*n*, %)	156 (3%)	152 (3%)	4 (3%)	0.79
	History of STI (*n*, %)	**937 (20%)**	**926 (20%)**	**11 (10%)**	**< 0.01**

CKD stage	Stage G3: 30 to 59 mL/min/1.73 m^2^ (*n*, %)	**346 (7%)**	**330 (7%)**	**16 (14%)**	**< 0.01**
	No CKD: 60 to 89 mL/min/1.73 m^2^ (*n*, %)	**1221 (26%)**	**1182 (26%)**	**39 (33%)**	
	No CKD: 90–120 mL/min/1.73 m^2^ (*n*, %)	**2738 (57%)**	**2679 (58%)**	**59 (50%)**	
	Hyperfiltration > 120 mL/min/1.73 m^2^ (*n*, %)	**445 (9%)**	**442 (10%)**	**3 (3%)**	

Socioeconomic status	Material deprivation index				
	1 (least deprived) (*n*, %)	**1470 (31%)**	**1433 (32%)**	**37 (32%)**	**< 0.01**
	2 (*n*, %)	**833 (18%)**	**815 (19%)**	**18 (15%)**	
	3 (*n*, %)	**764 (16%)**	**744 (16%)**	**20 (17%)**	
	4 (*n*, %)	**788 (17%)**	**769 (15%)**	**19 (16%)**	
	5 (most deprived) (*n*, %)	**895 (19%)**	**872 (18%)**	**23 (20%)**	

Medications	ACE inhibitor (*n*, %)	**153 (3%)**	**141 (3%)**	**12 (10%)**	**0.01**
	ARB (*n*, %)	**81 (2%)**	**76 (2%)**	**5 (4%)**	**0.04**
	SGLT-2 inhibitor (*n*, %)	30 (0.6%)	30 (0.7%)	0 (0%)	N/A
	COX-1 inhibitor (*n*, %)	553 (12%)	533 (11%)	20 (17%)	0.08
	COX-2 inhibitor (*n*, %)	**26 (0.5%)**	**23 (0.5%)**	**3 (2%)**	**0.03**

	Follow-up time in years (median, IQR)	**1.0 (1.9)**	**1.0 (1.9)**	**0.3 (1.3)**	**< 0.01**

	Number of HIV tests in follow-up	**4.9 (4.9)**	**5.0 (4.9)**	**2.4 (2.3)**	**< 0.01**

*Note:* Bold signifies *p* < 0.05.

Abbreviations: ACE = angiotensin-converting enzyme, ARB = angiotensin receptor blocker, CKD = chronic kidney disease, COX = cyclooxygenase, SD = standard deviation, SGLT-2 = sodium-glucose transporter 2, TAF = tenofovir alafenamide, and TDF = tenofovir disoproxil fumarate.

**Table 2:** Cohort baseline demographics by eGFR/CKD stage.

		**eGFR > 120 mL/min/1.73 ** **m** ^2^ **hyperfiltration (*n* = 445)**	**eGFR 90–120 mL/min/1.73 ** **m** ^2^ **no CKD (*n* = 2738)**	**eGFR 60–89 mL/min/1.73 ** **m** ^2^ **no CKD (*n* = 1221)**	**eGFR < 60 mL/min/1.73 ** **m** ^2^ **CKD stage G3 (*n* = 346)**	**p** **value**

Demographics	Age (mean, SD)	**25.1 (5)**	**33.6 (9)**	**42.0 (12)**	**46.6 (11)**	**< 0.01**
	Female sex (*n*, %)	**41 (9%)**	**164 (6)**	**79 (6)**	**104 (31)**	**< 0.01**

Geography	Urban	**401 (90%)**	**2586 (94)**	**1159 (95)**	**321 (93)**	**< 0.01**

Comorbidities	Hypertension (*n*, %)	**15 (3)**	**166 (6)**	**138 (11)**	**58 (17)**	**< 0.01**
	Diabetes (*n*, %)	**10 (2)**	**92 (3)**	**65 (5)**	**27 (8)**	**< 0.01**
	Heart failure (*n*, %)	1 (0.2)	5 (0.2)	3 (0.2)	3 (1)	0.12
	Stroke (*n*, %)	**1 (0.2)**	**3 (0)**	**7 (0.5)**	**4 (1)**	**< 0.01**
	Myocardial infarction (*n*, %)	**0 (0)**	**2 (0)**	**1 (0)**	**5 (1)**	**< 0.01**
	Alcohol misuse (*n*, %)	**11 (3)**	**29 (1)**	**13 (1)**	**15 (4)**	**< 0.01**
	Depression (*n*, %)	57 (13)	317 (12)	148 (12)	37 (11)	0.78
	Drug use (*n*, %)	**17 (4)**	**79 (3)**	**33 (3)**	**27 (8)**	**< 0.01**
	History of STI (*n*, %)	**91 (20)**	**565 (21)**	**258 (21)**	**23 (7)**	**< 0.01**

Socioeconomic status	Material deprivation index					
	1 (least deprived) (*n*, %)	**110 (24)**	**889 (32)**	**403 (33)**	**68 (19)**	**< 0.01**
	2 (*n*, %)	**81 (18)**	**489 (18)**	**227 (19)**	**36 (10)**	
	3 (*n*, %)	**76 (17)**	**436 (16)**	**197 (16)**	**55 (16)**	
	4 (*n*, %)	**82 (18)**	**449 (16)**	**181 (15)**	**76 (22)**	
	5 (most deprived) (*n*, %)	**96 (21)**	**475 (17)**	**213 (17)**	**111 (32)**	

Medications	ACE inhibitor (*n*, %)	**4 (1)**	**65 (2)**	**60 (5)**	**24 (7)**	**< 0.01**
	ARB (*n*, %)	**1 (0)**	**37 (1)**	**28 (2)**	**15 (4)**	**< 0.01**
	SGLT-2 inhibitor (*n*, %)	**0**	**14 (0.5)**	**15 (1)**	**1 (0)**	**< 0.01**
	COX-1 inhibitor (*n*, %)	**53 (12)**	**291 (10)**	**155 (13)**	**54 (16)**	**0.03**
	COX-2 inhibitor (*n*, %)	**0**	**11 (0.4)**	**10 (0.8)**	**5 (1)**	**0.02**

	TDF/FTC	**442 (99)**	**2679 (98)**	**1182 (97)**	**330 (95)**	**< 0.01**

	TAF/FTC	**3 (1)**	**59 (2)**	**39 (3)**	**16 (5)**	**< 0.01**

	Follow-up time in years (median, IQR)	**0.6 (1.4)**	**0.9 (1.8)**	**1.1 (2.1)**	**1.3 (1.8)**	**< 0.01**

	Number of HIV tests in follow-up	**3.8 (4.2)**	**4.9 (4.7)**	**5.7 (5.3)**	**4.4 (4.9)**	**< 0.01**

*Note:* Bold signifies *p* < 0.05.

Abbreviations: ACE = angiotensin-converting enzyme, ARB = angiotensin receptor blocker, CKD = chronic kidney disease, COX = cyclooxygenase, eGFR = estimated glomerular filtration, SD = standard deviation, SGLT-2 = sodium-glucose transporter 2, TAF = tenofovir alafenamide, and TDF = tenofovir disoproxil fumarate.

**Table 3:** Cox PH model for HIV infection risk factors with negative HIV serology within 30 days of index date.

	HR	95% CI	*p* value
Age (per 1 year increase)	**1.05**	**1.01, 1.09**	**0.01**
Male sex	**0.26**	**0.09, 0.76**	**0.01**
PrEP type (TAF/FTC = reference)	(Reference)	(Reference)	(Reference)
TDF/FTC	**0.26**	**0.08, 0.88**	**0.03**
Geography (rural = reference)	(Reference)	(Reference)	(Reference)
Urban	**0.21**	**0.08, 0.57**	**< 0.01**
CKD stage/eGFR (eGFR 90–120 mL/min/1.73 m^2^ = reference)	(Reference)	(Reference)	(Reference)
Stage G3 < 60 mL/min/1.73 m^2^	2.00	0.64, 6.28	0.24
No CKD eGFR 60–89 mL/min/1.73 m^2^	0.32	0.10, 1.04	0.06
Hyperfiltration > 120 mL/min/1.73 m^2^	1.14	0.23, 5.59	0.88
Depression	0.75	0.22, 2.59	0.65
Material deprivation index (1st quintile = reference)	(Reference)	(Reference)	(Reference)
2nd quintile	1.11	0.35, 3.45	0.86
3rd quintile	0.21	0.03, 1.70	0.14
4th quintile	0.90	0.28, 2.83	0.85
5th quintile	1.43	0.51, 4.00	0.49
Drug use	3.65	0.80, 16.68	0.09
History of sexually transmitted infection	1.32	0.55, 3.20	0.54

*Note: n* = 3579, 30 events. Bold signifies *p* < 0.05.

Abbreviations: CI = confidence interval, CKD = chronic kidney disease, eGFR = estimated glomerular filtration rate, HR = hazard ratio, PrEP = pre-exposure prophylaxis, TAF = tenofovir alafenamide, and TDF = tenofovir disoproxil fumarate.

**Table 4:** Cox PH model for HIV infection risk factors, among those with negative HIV serology within −180 days of index date.

	HR	95% CI	*p* value
Age (per 1 year increase)	**1.04**	**1.02, 1.06**	**< 0.01**
Male sex	**0.24**	**0.14, 0.41**	**< 0.01**
PrEP type (TAF/FTC = reference)	(Reference)	(Reference)	(Reference)
TDF/FTC	0.55	0.24, 1.25	0.15
Geography (rural = reference)	(Reference)	(Reference)	(Reference)
Urban	0.59	0.29, 1.18	0.14
CKD stage/eGFR (eGFR 90–120 mL/min/1.73 m^2^ = reference)	(Reference)	(Reference)	(Reference)
Stage G3 < 60 mL/min/1.73 m^2^	**3.27**	**1.86, 5.73**	**< 0.01**
No CKD eGFR 60–89 mL/min/1.73 m^2^	**0.44**	**0.22, 0.89**	**0.02**
Hyperfiltration > 120 mL/min/1.73 m^2^	1.50	0.56, 3.99	0.42
Depression	0.44	0.19, 1.01	0.05
Material deprivation index (1st quintile = reference)	(Reference)	(Reference)	(Reference)
2nd quintile	1.18	0.58, 2.43	0.65
3rd quintile	0.89	0.41, 1.92	0.77
4th quintile	1.24	0.64, 2.41	0.52
5th quintile	1.34	0.71, 2.56	0.37
Drug use	**3.41**	**1.53, 7.64**	**< 0.01**
History of sexually transmitted infection	0.62	0.31, 1,24	0.17

*Note: n* = 4071, 84 events. Bold signifies *p* < 0.05.

Abbreviations: CI = confidence interval, CKD = chronic kidney disease, eGFR = estimated glomerular filtration rate, HR = hazard ratio, PrEP = pre-exposure prophylaxis, TAF = tenofovir alafenamide, and TDF = tenofovir disoproxil fumarate.

**Table 5:** Cox PH model for HIV infection risk factors, among those without an HIV ICD-9/10 code or positive HIV serology −365 days of index date.

	HR	95% CI	*p* value
Age (per 1 year increase)	**1.04**	**1.03, 1.05**	**< 0.01**
Male sex	**0.34**	**0.27, 0.44**	**< 0.01**
PrEP type (TAF/FTC = reference)	(Reference)	(Reference)	(Reference)
TDF/FTC	1.38	0.72, 2.66	0.33
Geography (rural = reference)	(Reference)	(Reference)	(Reference)
Urban	0.89	0.63, 1.27	0.53
CKD stage/eGFR (eGFR 90–120 mL/min/1.73 m^2^ = reference)	(Reference)	(Reference)	(Reference)
Stage G3 < 60 mL/min/1.73 m^2^	**2.38**	**1.82, 3.14**	**< 0.01**
No CKD eGFR 60–89 mL/min/1.73 m^2^	**0.58**	**0.41, 0.81**	**< 0.01**
Hyperfiltration > 120 mL/min/1.73 m^2^	0.99	0.54, 1.81	0.97
Depression	0.77	0.53, 1.12	0.17
Material deprivation index (1st quintile = reference)	(Reference)	(Reference)	(Reference)
2nd quintile	0.86	0.55, 1.35	0.50
3rd quintile	1.23	0.83, 1.83	0.30
4th quintile	**1.68**	**1.18, 2.40**	**< 0.01**
5th quintile	**2.26**	**1.63, 3.14**	**< 0.01**
Drug use	**2.11**	**1.45, 3.08**	**< 0.01**
History of sexually transmitted infection	**0.45**	**0.29, 0.72**	**< 0.01**

*Note: n* = 4750, 335 events. Bold signifies *p* < 0.05.

Abbreviations: CI = confidence interval, CKD = chronic kidney disease, eGFR = estimated glomerular filtration rate, HR = hazard ratio, PrEP = pre-exposure prophylaxis, TAF = tenofovir alafenamide, and TDF = tenofovir disoproxil fumarate.

**Table 6:** Logistic regression model for HIV infection risk factors.

	OR	95% CI	*p* value
Age (per 1 year increase)	**1.05**	**1.04, 1.06**	**< 0.01**
Male sex	**0.19**	**0.14, 0.26**	**< 0.01**
PrEP type (TAF/FTC = reference)	(Reference)	(Reference)	(Reference)
TDF/FTC	**2.25**	**1.08, 5.18**	**0.04**
Geography (rural = reference)	(Reference)	(Reference)	(Reference)
Urban	0.65	0.42, 1.02	0.05
CKD stage/eGFR (eGFR 90–120 mL/min/1.73 m^2^ = reference)	(Reference)	(Reference)	(Reference)
Stage G3 < 60 mL/min/1.73 m^2^	**5.09**	**3.69, 7.03**	**< 0.01**
No CKD eGFR 60–89 mL/min/1.73 m^2^	**0.56**	**0.38, 0.81**	**< 0.01**
Hyperfiltration > 120 mL/min/1.73 m^2^	0.73	0.38, 1.33	0.33
Depression	**0.61**	**0.38, 0.94**	**0.03**
Material deprivation index (1st quintile = reference)	(Reference)	(Reference)	(Reference)
2nd quintile	0.78	0.47, 1.28	0.34
3rd quintile	1.12	0.71, 1.75	0.59
4th quintile	**1.79**	**1.02, 2.67**	**0.01**
5th quintile	**2.37**	**1.65, 3.43**	**< 0.01**
Drug use	**2.51**	**1.48, 4.20**	**< 0.01**
History of sexually transmitted infection	**0.37**	**0.22, 0.59**	**< 0.01**

*Note: n* = 4750, 335 events. Bold signifies *p* < 0.05.

Abbreviations: CI = confidence interval, CKD = chronic kidney disease, eGFR = estimated glomerular filtration rate, HR = hazard ratio, PrEP = pre-exposure prophylaxis, TAF = tenofovir alafenamide, and TDF = tenofovir disoproxil fumarate.

## Data Availability

The data that support the findings of this study are available upon request from the corresponding author. The data are not publicly available due to privacy or ethical restrictions.

## Supporting Information

The appendix contains tables, a figure, and the STROBE checklist.
