# Supplementary material for: The Real-World Effectiveness of Human Immunodeficiency Virus Pre-Exposure Prophylaxis in Adults in Alberta, Canada: A Retrospective Population-Based Cohort Study
Source: Can J Infect Dis Med Microbiol. 2025 Aug 10;2025:9340622. doi: 10.1155/cjid/9340622 (PMC12358228; doi:10.1155/cjid/9340622)
Supplement: Supporting Information — Additional supporting information can be found online in the Supporting Information section. [file 9340622.f1.zip › Appendix (Tables S1-S6) v1.0 2025-06-04 clean.docx]

## Appendix Table 1: Codes to Identify Eligibility Criteria

| **Hospital/Physician Claims** | **ICD-9 Codes/Tariffs** | **ICD-10 Codes/Tariffs** |
| --- | --- | --- |
| HIV | 42 | B20, B21, B22, B23, B24 |
| HBV | [070.2](http://www.icd9data.com/2015/Volume1/001-139/070-079/070/070.2.htm), [070.20](http://www.icd9data.com/2015/Volume1/001-139/070-079/070/070.20.htm), 070.21, 070.22,  070.23, 070.3, 070.31, 070.32, 070.33 | B16, B16.0, B16.1, B16.2, B16.9, B18.0 B18.1, B19.1, B19.10, B19.11 |
| Transplant | -At least 1 hospitalization or physician claim for kidney transplant:  ICD-9-CM procedure: 55.6; physician billing code: 13.99Y, 03.04O, 03.04P | - At least 1 hospitalization or physician claim for kidney transplant: ICD-10-CA intervention: 1PC85, 1OK85, physician billing code: 13.99Y, 03.04O, 03.04P |
| Dialysis | Physician billing codes: 13.99A, 13.99B, 13.99C, 13.99D, 13.99OA, 13.99O, 13.99AB | Physician billing codes: 13.99A, 13.99B, 13.99C, 13.99D, 13.99OA, 13.99O, 13.99AB |

## Appendix Table 2: List of Required Databases

| **Database** | **Years** | **Data Fields** |
| --- | --- | --- |
| Laboratory  Provincial Laboratory Database and Connect Care | 2009-present | Scrambled ULI  Lab Results – Chemistry  Serum Creatinine  Blood Urea Nitrogen  Bicarbonate  Hemoglobin A1C  Urinalysis  (pH, glucose, protein)  Urine ACR  Urine PCR  24 hour urine for proteinuria  HIV antibody  HIV RNA  HBsAg  HBsAb  HBcAb  HBeAg  HBV DNA  Gonorrhea NAT  Chlamydia NAT  Syphilis EIA  Date of Collection  Location of Test  Test Description  Test Numeric Results |
| Inpatient: Discharge Abstract Database/Hospital Morbidity | 1993-present | Scrambled ULI  Admission Date  Separation Date  Diagnosis Codes  Intervention Codes  Intervention Dates  Length of Stay |
| Population Registry | 1993-present | Scrambled ULI  Sex  Date of Birth  Coverage Dates (start/end)  Reason for Coverage Cancellation  Postal Code  Municipality Code  Pampalon for Material Deprivation Index (2016) |
| Practitioner Claims | 1994-present | Scrambled ULI  Date of Service  Tariff Code  Diagnosis Code  Specialty Sub-bloc  Fee Charged |
| Pharmaceutical Information Network (PIN) Dispenses | 2008-present | Scrambled ULI  Date Prescription Provided  Length of Prescription (Days Supplied)  DIN  Dosage  ATC Code  Drug Cost Claimed  Drug Cost Paid  Product Description  Product Name |
| Ambulatory Care  (National Ambulatory Care Reporting System) | 1997-present | Scrambled ULI  Emergency department visits  Day procedures |
| Vital Statistics | 1999-present | Scrambled ULI  Death |

##

## Appendix Table 3: Exposures of Interest (Medications)

| Class | ATC code | Name |
| --- | --- | --- |
| PrEP | J05AR03 | TDF/FTC |
|  | J05AR17 | TAF/FTC |
| ACE inhibitor | C09AA01 | [Captopril](https://www.whocc.no/atc_ddd_index/?code=C09AA01&showdescription=yes) |
|  | C09AA02 | [Enalapril](https://www.whocc.no/atc_ddd_index/?code=C09AA02&showdescription=yes) |
|  | C09AA03 | [Lisinopril](https://www.whocc.no/atc_ddd_index/?code=C09AA03&showdescription=yes) |
|  | C09AA04 | [Perindopril](https://www.whocc.no/atc_ddd_index/?code=C09AA04&showdescription=yes) |
|  | C09AA05 | [Ramipril](https://www.whocc.no/atc_ddd_index/?code=C09AA05&showdescription=yes) |
|  | C09AA06 | [Quinapril](https://www.whocc.no/atc_ddd_index/?code=C09AA06&showdescription=yes) |
|  | C09AA07 | [Benazepril](https://www.whocc.no/atc_ddd_index/?code=C09AA07&showdescription=yes) |
|  | C09AA08 | [Cilazapril](https://www.whocc.no/atc_ddd_index/?code=C09AA08&showdescription=yes) |
|  | C09AA09 | [Fosinopril](https://www.whocc.no/atc_ddd_index/?code=C09AA09&showdescription=yes) |
|  | C09AA10 | [Trandolapril](https://www.whocc.no/atc_ddd_index/?code=C09AA10&showdescription=yes) |
| ARB | C09CA01 | [Losartan](https://www.whocc.no/atc_ddd_index/?code=C09CA01&showdescription=yes) |
|  | C09CA03 | [Valsartan](https://www.whocc.no/atc_ddd_index/?code=C09CA03&showdescription=yes) |
|  | C09CA04 | Irbesartan |
|  | C09CA06 | C[andesartan](https://www.whocc.no/atc_ddd_index/?code=C09CA06&showdescription=yes) |
|  | C09CA07 | [Telmisartan](https://www.whocc.no/atc_ddd_index/?code=C09CA07&showdescription=yes) |
|  | C09CA08 | Olmesartan |
| SGLT 2 inhibitor | A10BK03 | Empagliflozin |
|  | A10BK01 | Dapagliflozin |
|  | A10BK02 | Canagliflozin |
|  | A10BK06 | Sotagliflozin |
|  | A10BK04 | Ertugliflozin |
| NSAID COX-1 | M01AB01 | Indomethacin |
|  | M01AB05 | Diclofenac |
|  | M01AB15 | Ketorolac |
|  | M01AE01 | Ibuprofen |
|  | M01AE02 | Naproxen |
| NSAID COX-2 | M01AH01 | Celecoxib |

## Appendix Table 4: Potential Confounders (Comorbidities)

| **Hospital/Physician Claims** | **ICD-9 Codes** | **ICD-10 Codes** |
| --- | --- | --- |
| **Hypertension** | 401–405 | I10-I13, I15 |
| **Diabetes** | 250 | E10-E14 |
| **Heart Failure** | 398.91, 402.01, 402.11, 402.91, 404.01, 404.03,  404.11, 404.13, 404.91, 404.93, 425.4–425.9,  428 | I09.9, I25.5, I42.0, I42.5–I42.9, I43, I50 |
| **Stroke/Transient Ischemic Attack** | 362.3, 430, 431, 433X1, 434X1, 435, 436 | G45.0-G45.3, G45.8-G45.9, H34.1, I60, I61, I63, I64 |
| **Myocardial infarction** | 410 | I21-I22 |
| **Peripheral vascular disease** | 440.2 | I70.2 |
| **Drug use** | 292.2.x, 304.x, 305.2-305.9, V65.42 | F11.x-F16.x, F18.x, F19.x, Z71.5, Z72.2 |
| **Gonorrhea** | 98.0x, 98.1x | A54, A54.0x, A54.1, A54.2x |
| **Chlamydia** | 99.41 99.53, 99.54, 99.55 | A55, A56, A71, A74.9 |
| **Syphilis** | 91-97 | A51-A53 |
| **Sexually Transmitted Infection** | 614.0, 614.2, 614.3, 614.5, 614.8, 614.9, 615.0, 615.9 and 595.0, 597.80, 597.89, 597.81, 599.0, 601.0, 601.3, 604.90, 604.99, 616.0, 616.10, 616.11, 788.1, 788.7 | N30.0x*, N34.1-N34.3, N39.0, N41.0, N41.3, N45.1-N45.3, N70.0x, N71.0, N72, N73.8, N73.9, N76.0, N76.2, N77.1, R30.0, R30.9, R36.0, R36.9 |

## Appendix Table 5: Cox PH model for HIV infection risk factors, among those without an HIV ICD-9/10 code or positive HIV serology -365 days of index date censored at last HIV test

|  | HR | 95% CI | P value |
| --- | --- | --- | --- |
| Age (per 1 year increase) | **1.04** | **1.03, 1.05** | **<0.01** |
| Male sex | **0.36** | **0.28, 0.46** | **<0.01** |
| PrEP type (TAF/FTC = reference)  TDF/FTC | (reference)  1.34 | (reference)  0.69, 2.58 | (reference)  0.37 |
| Geography (rural = reference)  Urban | (reference)  0.89 | (reference)  0.62, 1.26 | (reference)  0.51 |
| CKD stage/eGFR (eGFR 90-120mL/min/1.73m^2^ = reference)  Stage G3 <60mL/min/1.73m^2^ No CKD eGFR 60-89mL/min/1.73m^2^ Hyperfiltration >120mL/min/1.73m^2^ | (reference)  **2.26** **0.56** 0.99 | (reference)  **1.72, 2.97 0.40, 0.79** 0.54, 1.83 | (reference)  **<0.01 <0.01** 0.98 |
| Depression | 0.77 | 0.53, 1.12 | 0.17 |
| Material Deprivation Index (1^st^ quintile = reference) 2^nd^ quintile 3^rd^ quintile 4^th^ quintile 5^th^ quintile | (reference)  0.87 1.24 **1.67 2.25** | (reference)  0.55, 1.36 0.83, 1.84 **1.17, 2.38 1.63, 3.13** | (reference)  0.54 0.29 **<0.01 <0.01** |
| Drug use | **2.10** | **1.44, 3.07** | **<0.01** |
| History of sexually transmitted infection | **0.46** | **0.29, 0.73** | **<0.01** |

n=4750, mean follow-up 1.29 years (SD 1.3), median follow-up 0.83 years (IQR 1.89), 335 events,

incident rate of 5.43 (95% CI 5.07, 5.82) HIV infections per 100 person years.

Note: PrEP=pre-exposure prophylaxis, TAF= tenofovir alafenamide, TDF=tenofovir disoproxil fumarate, CKD=chronic kidney disease, eGFR=estimated glomerular filtration rate, HR=hazard ratio, CI=confidence interval

## Appendix Table 6: HIV Incidence Rate by Each Subgroup

| Subgroup | HIV incident rate/100 person years (95% CI) |
| --- | --- |
| Age <=25 | 1.11 (0.90, 1.36) |
| Age >25 | 5.45 (5.05, 5.88) |
| Male sex | 3.39 (3.14, 3.66) |
| Female sex | 19.80 (16.40, 23.80) |
| Urban | 4.59 (4.27, 4.94) |
| Rural | 9.56 (7.42, 12.3) |
| eGFR>120mL/min/1.73m^2^ | 2.48 (1.90, 3.24) |
| eGFR 90-120mL/min/1.73m^2^ | 3.44 (3.11, 3.80) |
| eGFR 60-89mL/min/1.73m^2^ | 2.62 (2.25, 3.05) |
| eGFR<60mL/min/1.73m^2^ | 20.70 (17.40, 24.70) |
| Material Deprivation Index 1^st^ quintile | 2.56 (2.23, 2.94) |
| Material Deprivation Index 2^nd^ quintile | 2.42 (2.01, 2.91) |
| Material Deprivation Index 3^rd^ quintile | 3.89 (3.25, 4.66) |
| Material Deprivation Index 4^th^ quintile | 6.85 (5.84, 8.04) |
| Material Deprivation Index 5^th^ quintile | 10.54 (9.16, 12.13) |
| History of depression | 4.30 (3.50, 5.27) |
| No history of depression | 4.95 (4.59, 5.33) |
| Drug use | 20.20 (15.57, 26.24) |
| Drug use | 4.51 (4.19, 4.84) |
| History of sexually transmitted infection | 5.65 (5.24, 6.09) |
| No history of sexually transmitted infection | 1.55 (1.28, 1.88) |

## Appendix Table 7: Contemporary PrEP Epidemiologic Studies

| Study name  (Author) | Country  (Year) | Design | PrEP type/regimen | Population  (% male) | Follow-up | HIV incidence  (per 100 patient years) | Effect size | Adherence |
| --- | --- | --- | --- | --- | --- | --- | --- | --- |
| ANRS PREVENIR^1^  (Molina) | France  (2017-2020) | Prospective observational cohort study | TDF/FTC daily or on-demand | 3065  1540 daily PrEP  1509 on-demand PrEP  (99.1%) | 1 month, q3 months with  median 2.21 months follow-up (IQR 15.9-29.7) | 0.11  95% CI 0.04-0.23 | N/A | Self-reported, dried blood spots |
| Demo Project^2^  (Liu) | United States  (2012-2015) | Prospective observational cohort study | TDF/FTC | 557  (98.4%) | 4,12,24,36,48 weeks | 0.43  95% CI 0.05-0.154 | N/A | Self-reported, pill counts, dried blood spots |
| Veterans’ Health Aministration^3^  (Van Epps) | United States (2012-2016) | Retrospective observational cohort study | TDF/FTC | 825  (97%) | Median 8 months | 0.8  95% CI 0.3-1.8 | N/A | Pharmacy dispensation and number of days in possession |
| Kaiser Permanente Norther California^4^  (Hojilla) | United States (2012-2019) | Retrospective observational cohort study | TDF/FTC or TDF-TAC | 13906  (95.1%) | Median 1.6 years (IQR 0.7-2.8) | 0.35  95% CI 0.28-0.43 | N/A | Not reported |
| Scotland National HIV Program^5^  (Estcourt) | Scotland  (2015-2019) | Retrospective observational cohort study  Pre/Post | TDF/FTC  Daily or event driven | 14319 Pre PreP  16723  Post PrEP  (100%) | Not reported | 0.174  95% CI 0.058-0.414 for PrEP | aIRR 0.25 95% CI 0.09-0.70 | Not reported |
| ^6^N/A  (Jourdain) | France  (2016-2020) | Matched nested case-control study | TDF/FTC | 256  cases  1213 controls  (100%) | Not reported | 0.22 | aOR 0.40 95% CI 0.29-0.54, varied by PrEP consumption | PreP consumption via number of days covered by PreP dispensing |
| National Evaluation of PrEP Outcomes (NEPOS)^7^ (Schmidt) | Germany  (2019-2020) | Retrospective observational cohort study | TDF/FTC  Daily or event-driven | 4620  3737 daily  874 event-driven  (99.2%) | Median 451 days (IQR 357-488) | 0.078  95% CI 0.029-0.208 | N/A | Pill coverage |
| China Real-World Oral Intake of PrEP (CROPrEP)^8^  (Wang) | China  (2018-2020) | Nonrandomized controlled trial | TDF/FTC Daily or event-driven | 2037  1027 daily  503 event driven (100%) | 1,3,6,9,12 months | 0.64  95% CI 0.26-1.31 | aIRR 0.09  95% CI 0.04-0.21 | Self-reported, dispensing, pill counts |

Note: PreP = pre-exposure prophylaxis, HIV = human immunodeficiency virus, IQR = interquartile range, TDF/FTC = tenofovir disoproxil fumarate, TAF/FTC = tenofovir alefenamide, aIRR = adjusted incident rate ratio, aOR = adjusted odds ratio

## Appendix Figure 1: Kaplan Meier Survival Curve


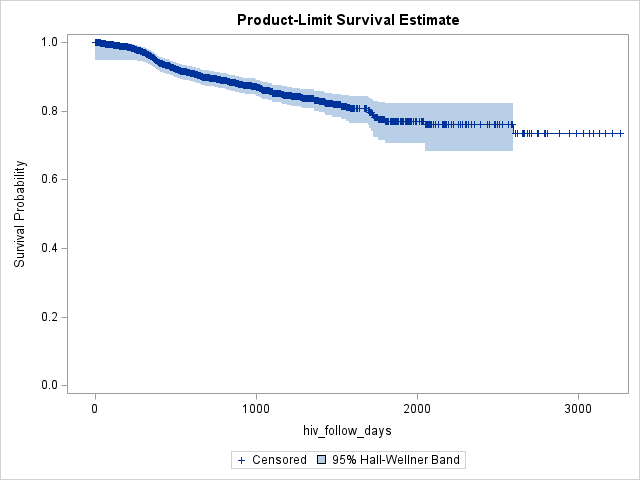


**References**

1. Molina JM, Ghosn J, Assoumou L, et al. Daily and on-demand HIV pre-exposure prophylaxis with emtricitabine and tenofovir disoproxil (ANRS PREVENIR): a prospective observational cohort study. *Lancet HIV*. Aug 2022;9(8):e554-e562. doi:10.1016/S2352-3018(22)00133-3

2. Liu AY, Cohen SE, Vittinghoff E, et al. Preexposure Prophylaxis for HIV Infection Integrated With Municipal- and Community-Based Sexual Health Services. *JAMA Intern Med*. Jan 2016;176(1):75-84. doi:10.1001/jamainternmed.2015.4683

3. Van Epps P, Wilson BM, Garner W, Beste LA, Maier MM, Ohl ME. Brief Report: Incidence of HIV in a Nationwide Cohort Receiving Pre-exposure Prophylaxis for HIV Prevention. *J Acquir Immune Defic Syndr*. Dec 15 2019;82(5):427-430. doi:10.1097/QAI.0000000000002186

4. Hojilla JC, Hurley LB, Marcus JL, et al. Characterization of HIV Preexposure Prophylaxis Use Behaviors and HIV Incidence Among US Adults in an Integrated Health Care System. *JAMA Netw Open*. Aug 2 2021;4(8):e2122692. doi:10.1001/jamanetworkopen.2021.22692

5. Estcourt C, Yeung A, Nandwani R, et al. Population-level effectiveness of a national HIV preexposure prophylaxis programme in MSM. *AIDS*. Mar 15 2021;35(4):665-673. doi:10.1097/QAD.0000000000002790

6. Jourdain H, de Gage SB, Desplas D, Dray-Spira R. Real-world effectiveness of pre-exposure prophylaxis in men at high risk of HIV infection in France: a nested case-control study. *Lancet Public Health*. Jun 2022;7(6):e529-e536. doi:10.1016/S2468-2667(22)00106-2

7. Schmidt D, Kollan C, Bartmeyer B, et al. Low incidence of HIV infection and decreasing incidence of sexually transmitted infections among PrEP users in 2020 in Germany. *Infection*. Jun 2023;51(3):665-678. doi:10.1007/s15010-022-01919-3

8. Wang H, Wang Z, Huang X, et al. Association of HIV Preexposure Prophylaxis Use With HIV Incidence Among Men Who Have Sex With Men in China: A Nonrandomized Controlled Trial. *JAMA Netw Open*. Feb 1 2022;5(2):e2148782. doi:10.1001/jamanetworkopen.2021.48782
